# Emotional support, education and self-rated health in 22 European countries

**DOI:** 10.1186/1471-2458-7-272

**Published:** 2007-10-01

**Authors:** Olaf von dem Knesebeck, Siegfried Geyer

**Affiliations:** 1 Department of Medical Sociology, University Medical Centre Hamburg-Eppendorf, Martinistr. 52, 20246 Hamburg, Germany; 2 Medical Sociology Unit, Hannover Medical School, Carl-Neuberg-Str. 1, 30625 Hannover, Germany

## Abstract

**Background:**

The analyses focus on three aims: (1) to explore the associations between education and emotional support in 22 European countries, (2) to explore the associations between emotional support and self-rated health in the European countries, and (3) to analyse whether the association between education and self-rated health can be partly explained by emotional support.

**Methods:**

The study uses data from the European Social Survey 2003. Probability sampling from all private residents aged 15 years and older was applied in all countries. The European Social Survey includes 42,359 cases. Persons under age 25 were excluded to minimise the number of respondents whose education was not complete. Education was coded according to the International Standard Classification of Education. Perceived emotional support was assessed by the availability of a confidant with whom one can discuss intimate and personal matters with. Self-rated health was used as health indicator.

**Results:**

Results of multiple logistic regression analyses show that emotional support is positively associated with education among women and men in most European countries. However, the magnitude of the association varies according to country and gender. Emotional support is positively associated with self-rated health. Again, gender and country differences in the association were observed. Emotional support explains little of the educational differences in self-rated health among women and men in most European countries.

**Conclusion:**

Results indicate that it is important to consider socio-economic factors like education and country-specific contexts in studies on health effects of emotional support.

## Background

There is considerable evidence that social support is beneficial to health. Studies have consistently shown an association of social support with mortality [1] and morbidity [2] as well as with self-rated health [3]. Social support refers to the quality and type of support provided by social network members. Social support is typically divided into subtypes, which include emotional and instrumental support [4,5]. Emotional support is most often provided by a confidant or intimate other and is related to understanding, esteem and help in decision making. Instrumental support may be manifested in many forms, including practical help and financial support. In some studies other subtypes of social support have been identified which may be allied to emotional support. Moreover, there is a conceptual differentiation between perceived and received social support. Perceived support relates to an individual's subjective appraisal of their satisfaction with support or their perception that support would be available if needed. Perceived emotional support (i.e. beliefs that love and caring, sympathy and understanding, and/or esteem and value are available from others) has been found a powerful determinant of health in epidemiological studies [6]. Measures capturing support that has actually been received have been used less frequently in studies of social determinants of health.

Studies on health effects of social support have been conducted in different countries, like the United States [7,8], France [3], Germany [9], Finland [1] or the United Kingdom [10]. However, there is a lack of international comparisons. Furthermore, only a few studies have examined how social support is linked to structural and economic conditions. The evidence that individuals with a low socio-economic position have relationships of lower quality is scattered and inconsistent. While a few studies have found that higher levels of social support are related to higher occupational positions [11-13] and higher income levels [14], less is known about the association between education and social support. Differentiating between these indicators is appropriate because occupational position, income and education cannot be used interchangeably [15]. Moreover, it is unclear whether the widely known socio-economic differences in health can be partly explained by effects of social support [5,9,16,17].

Against this background, the following analyses focus on three aims: (1) to explore the associations between education and emotional support in 22 European countries, (2) to explore the associations between emotional support and self-rated health in the European countries, and (3) to analyse whether the association between education and self-rated health can be partly explained by emotional support.

## Methods

### Study population

The analyses are based on the first wave of the European Social Survey [18,19]. Data from face to face interviews were available from 22 countries: Austria, Belgium, Czech Republic, Denmark, Finland, France, Germany, Greece, Hungary, Ireland, Israel (the only non-European country), Italy, Luxembourg, the Netherlands, Norway, Poland, Portugal, Slovenia, Spain, Sweden, Switzerland, and the United Kingdom. Probability sampling from all private residents aged 15 years and older was applied in all countries. The European Social Survey includes 42,359 cases. Average response rate is about 60.6%, ranging from 33.5% in Switzerland to 80.0% in Greece [19,20]. Because we use educational attainment as a measure of socio-economic position, we exclude persons under age 25 to minimise the number of respondents whose education was not complete at the time of the interview. This restriction results in a sample size of 36,263 participants. Response rates, numbers of remaining cases for each country and the distribution of the study variables are shown in table 1.

**Table 1:** Description of the study population and variables (European Social Survey, weighted, N = 36,263)

Country (response rate in %; N)	Education (upper secondary or higher, %)		Emotional support (existence of a confidant, %)		Self-rated health (good or very good, %)	
	Men	Women	Men	Women	Men	Women

Austria (60.4; 1,993)	70.5	68.3	90.3	91.8	76.6	74.7
Belgium (59.2; 1,511)	67.8	61.9	86.2	85.7	78.6	73.7
Czech Rep. (43.3; 1,183)	93.8	81.4	85.9	85.3	57.2	46.1
Denmark (67.5; 1,301)	82.1	76.7	91.7	94.2	78.7	75.6
Finland (73.2; 1,670)	61.8	62.2	87.2	92.7	63.2	62.1
France (43.1; 1,321)	46.9	43.8	86.0	88.1	61.5	55.9
Germany (57.1; 2,525)	94.9	85.2	94.5	96.5	57.7	55.5
Greece (80.0; 2,290)	44.6	36.1	91.5	90.9	77.2	65.6
Hungary (69.9; 1,436)	37.2	38.5	90.9	92.6	42.7	35.4
Ireland (64.5; 1,753)	52.4	53.9	90.1	93.1	84.7	81.9
Israel (71.0; 1,920)	79.0	82.3	90.2	91.8	72.3	65.7
Italy (43.7; 1,073)	44.3	40.5	74.3	80.9	66.8	56.4
Luxembourg (43.9; 1,182)	65.9	56.0	84.6	90.4	69.1	59.3
Netherlands (67.9; 2,170)	62.8	52.4	92.9	94.6	76.3	71.8
Norway (65.0; 1,810)	84.3	83.0	94.9	96.4	76.5	70.0
Poland (73.2; 1,639)	40.5	47.6	86.8	87.5	53.2	42.2
Portugal (68.8; 1,312)	20.8	20.2	90.5	88.7	54.1	38.8
Sweden (69.5; 1,713)	51.0	51.6	89.2	93.3	74.9	68.4
Slovenia (70.5; 1,252)	77.1	65.6	90.9	86.7	58.5	44.2
Spain (53.2; 1,517)	42.8	37.8	89.6	90.7	65.9	57.1
Switzerland (33.5; 1,860)	89.3	81.9	96.7	95.3	86.3	81.3
UK (60.6; 1,832)	48.6	43.3	89.4	93.5	72.5	71.3

### Variables

Education was coded according to the International Standard Classification of Education (ISCED-97) [21]. Respondent's highest level of education was classified ranging from "not completed primary education" to "second stage of tertiary education" on a 7-point scale. The subjects were divided into two groups: (1) lower secondary, second stage of basic education, primary education, first stage of basic education or not completed primary education; (2) (upper) secondary, post secondary, first stage of tertiary or second stage of tertiary education. 175 respondents with missing value on the education variable (all countries together) were excluded. Table 1 shows marked differences in educational levels between the countries under study (see also [20]). In the Czech Republic, Denmark, Germany, Norway, and Switzerland more than 80% of the male respondents have an upper secondary or higher education, in Portugal the rate is about 20%. In most countries men have higher rates of high education.

Analyses are focussed on perceived emotional support as one of the most important dimensions of social support [6]. Perceived emotional support was assessed by the availability of a confidant with whom one can discuss intimate and personal matters with (yes or no). This measure was found to be a powerful predictor of health in other studies [22,23]. Table 1 shows that a confidant is available for most of the male and female respondents in all countries with values ranging from 74.3% (men, Italy) to 96.7% (men, Switzerland). Taking all countries together, 283 respondents have missing values on emotional support. These were excluded from the analyses.

We use a health indicator that has been found to be appropriate for comparative studies on social determinants of health: self-rated health [24,25]. Self-rated health reflects how respondents rate their health, answering a single-item on a 5-point scale ranging from "very good" (1) to "very bad" (5). It was shown that such self-ratings represent a source of reliable and valid data on health status [26,27]. Responses were dichotomised with "good" and "very good" indicating a favourable general health status. 36 cases with missing values on self-rated health (all countries) were excluded. As can be seen in table 1 there are cross-national variations in self-rated health (see also [20]). In Ireland and Switzerland more than 80% of the male and female respondents rate their health as "good" or "very good", whereas in Hungary the proportion is less than 50%. Moreover, in all countries men report a better health than women.

### Statistical methods

In all analyses, a design weight is applied to correct for slightly different probabilities of selection in some countries. To test associations between education, emotional support and self-rated health, multiple logistic regression analyses are conducted for each country. Odds ratios (OR) and 95% confidence intervals (CI) are displayed. Statistically significant OR (p < 0.05) are italicised. Adjustments are made for age. As several studies suggest that the effects of education and social support on health differ for men and women [5,20], separate analyses are conducted. To analyse whether the association between education and self-rated health can be explained by emotional support, two regression models are calculated. In the first model, we calculate an age adjusted OR for the relation between education and health. We then introduce emotional support into the model and compare age adjusted with age and emotional support adjusted results by quantifying the percentage change in OR [28]. Changes are calculated by using ([OR_age adjusted_-OR_age and emotional support adjusted_]/[OR_age adjusted_-1])*100. Percentage change is displayed when OR is statistically significant (p < 0.05) in the age adjusted model. All analyses are conducted with the statistical programme package SPSS 13.0.

## Results

### Education and emotional support

Table 2 shows the associations between education and emotional support in the 22 European countries for men and women. In general, people with high (upper secondary or higher) education have a higher probability of the existence of a confidant. Relationships are particularly strong (OR > 3.0) among men in Germany and Spain and among women in Austria and Israel. Associations are not significant for either sex in the Scandinavian countries (Denmark, Finland, Norway, and Sweden) and in Portugal. Associations are statistically significant in 10 countries among men and in 12 countries among women.

**Table 2:** Education (upper secondary or higher) and emotional support (adjusted for age): odds ratios* (95% confidence interval), European Social Survey, weighted

Country	Men (n = 16,763)	Women (n = 19,006)
Austria	*2.85 (1.79–4.53)*	*3.02 (1.85–4.91)*
Belgium	1.38 (0.89–2.14)	*1.71 (1.07–2.73)*
Czech Rep.	*2.80 (1.28–4.15)*	*1.91 (1.09–3.35)*
Denmark	1.53 (0.80–2.93)	1.03 (0.48–2.20)
Finland	1.53 (0.97–2.41)	1.38 (0.75–2.54)
France	*2.43 (1.42–4.17)*	1.71 (0.99–2.95)
Germany	*5.03 (2.54–9.95)*	1.68 (0.83–3.43)
Greece	0.65 (0.39–1.09)	*1.65 (1.02–2.68)*
Hungary	*2.28 (1.21–4.30)*	1.63 (0.85–3.11)
Ireland	1.62 (0.96–2.73)	*2.52 (1.41–4.51)*
Israel	1.54 (0.91–2.61)	*3.12 (1.86–5.24)*
Italy	*2.37 (1.48–3.78)*	*1.64 (1.00–2.70)*
Luxembourg	*2.24 (1.38–3.65)*	1.59 (0.86–2.95)
Netherlands	*1.69 (1.00–2.84)*	*1.89 (1.02–3.51)*
Norway	0.98 (0.46–2.10)	2.04 (0.85–4.86)
Poland	1.40 (0.90–2.19)	*2.02 (1.25–3.27)*
Portugal	1.71 (0.68–4.26)	1.39 (0.65–2.95)
Spain	*4.90 (2.79–10.59)*	1.81 (0.85–3.86)
Sweden	1.43 (0.90–2.28)	1.80 (0.92–3.53)
Slovenia	*2.20 (1.16–4.17)*	*2.38 (1.43–3.94)*
Switzerland	2.37 (0.96–5.83)	*2.30 (1.16–4.55)*
UK	1.07 (0.67–1.70)	*1.89 (1.01–3.54)*

* Statistically significant odds ratios (p < 0.05) are italicised.

### Emotional support and self-rated health

Emotional support is significantly associated with self-rated health in 12 countries among men (Table 3). Association is particularly strong in the Netherlands, where men who have a person with whom they can discuss personal or intimate matters have an almost threefold probability of reporting good or very good health. Among women, we find significant relationships in 11 countries. These associations are strong (OR > 3.0) in the Czech Republic, Germany, and Spain. Emotional support is not significantly related to self-rated health among men and women in Greece, Norway, Slovenia, and Switzerland.

**Table 3:** Emotional support and good or very good self-rated health (adjusted for age): odds ratios* (95% confidence interval), European Social Survey, weighted

Country	Men (n = 16,832)	Women (n = 19,094)
Austria	*1.78 (1.08–2.92)*	1.55 (0.94–2.55)
Belgium	*1.61 (1.01–2.57)*	*2.78 (1.78–4.36)*
Czech Rep.	1.65 (0.97–2.82)	*3.13 (1.67–5.87)*
Denmark	1.19 (0.63–2.25)	*2.55 (1.27–5.12)*
Finland	*1.62 (1.04–2.51)*	*1.85 (1.01–3.40)*
France	*2.22 (1.37–3.65)*	1.47 (0.88–2.43)
Germany	*2.05 (1.24–3.40)*	*4.57 (2.22–9.43)*
Greece	1.03 (0.56–1.88)	1.04 (0.67–1.61)
Hungary	*2.56 (1.33–4.96)*	1.43 (0.74–2.77)
Ireland	1.24 (0.66–2.30)	*1.99 (1.12–3.56)*
Israel	1.51 (0.90–2.52)	*2.12 (1.22–3.68)*
Italy	*2.08 (1.32–3.29)*	1.07 (0.67–1.69)
Luxembourg	*2.17 (1.32–3.55)*	1.68 (0.96–2.94)
Netherlands	*2.96 (1.75–5.01)*	1.35 (0.78–2.32)
Norway	1.47 (0.79–2.74)	1.32 (0.60–2.89)
Poland	1.30 (0.83–2.04)	*2.23 (1.32–3.77)*
Portugal	*2.26 (1.14–4.49)*	*2.21 (1.18–4.16)*
Spain	1.44 (0.82–2.53)	*3.14 (1.70–5.77)*
Sweden	*1.70 (1.07–2.70)*	*1.93 (1.07–3.50)*
Slovenia	1.60 (0.88–2.92)	1.72 (0.99–2.98)
Switzerland	1.36 (0.54–3.44)	1.80 (0.90–3.60)
UK	*1.92 (1.19–3.09)*	1.65 (0.94–2.87)

* Statistically significant odds ratios (p < 0.05) are italicised.

### Education, emotional support and self-rated health

To examine, whether the association between education and self-rated health is mediated by emotional support, self-rated health is regressed first on education controlling for age. Then self-rated health is regressed on education controlling for age and emotional support. Results documented in table 4 show, that men with high education have elevated probabilities of reporting good or very good health in most countries after adjustment for age [20]. These associations are only slightly attenuated after additional control for emotional support. There are only a few countries in which adjustment for emotional support leads to a reduction of more than 10% in the association between education and health (France, Italy, and Luxembourg). Thus, emotional support explains little of the educational differences in self-rated health among men in most countries.

**Table 4:** Education (upper secondary or higher) and good or very good self-rated health: odds ratios (OR)* (95% confidence interval), men, European Social Survey, weighted (n = 16,746)

Country	Adjusted for age	Adjusted for age and emotional support	Change (%)**
Austria	1.05 (0.73–1.49)	0.97 (0.68–1.40)	
Belgium	*1.65 (1.22–2.40)*	*1.62 (1.12–2.37)*	-5
Czech Rep.	0.99 (0.43–2.25)	0.89 (0.39–2.06)	
Denmark	1.28 (0.80–2.04)	1.27 (0.80–2.03)	
Finland	*1.92 (1.39–2.66)*	*1.89 (1.36–2.62)*	-3
France	*1.65 (1.15–2.35)*	*1.54 (1.08–2.21)*	-17
Germany	1.47 (0.85–2.52)	1.32 (0.76–2.28)	
Greece	*1.90 (1.25–2.87)*	*1.90 (1.26–2.88)*	0
Hungary	*2.12 (1.48–3.03)*	*2.05 (1.43–2.93)*	-6
Ireland	*2.26 (1.43–3.58)*	*2.25 (1.42–3.56)*	-1
Israel	*1.80 (1.22–2.64)*	*1.77 (1.20–2.60)*	-4
Italy	*1.56 (1.01–2.41)*	1.43 (0.92–2.20)	-23
Luxembourg	*2.11 (1.42–3.15)*	*1.97 (1.31–2.95)*	-13
Netherlands	*2.45 (1.77–3.38)*	*2.39 (1.73–3.31)*	-4
Norway	1.38 (0.92–2.07)	1.38 (0.92–2.07)	
Poland	*2.24 (1.63–3.08)*	*2.23 (1.62–3.06)*	-1
Portugal	*3.00 (1.78–5.04)*	*2.94 (1.79–4.96)*	-3
Spain	*1.52 (1.02–2.26)*	*1.48 (1.00–2.21)*	-8
Sweden	1.20 (0.87–1.70)	1.18 (0.85–1.64)	
Slovenia	*1.78 (1.16–2.72)*	*1.71 (1.14–2.62)*	-9
Switzerland	1.68 (0.96–2.92)	1.66 (0.95–2.89)	
UK	1.12 (0.81–1.54)	1.12 (0.81–1.55)	

* Statistically significant OR (p < 0.05) are italicised.

** Percentage change in OR in age adjusted analyses compared with OR in age and emotional support adjusted analyses. Calculated by using:

([OR_age adjusted_-OR_age and emotional support adjusted_]/[OR_age adjusted_-1])*100. Percentage change is displayed when OR is statistically significant in the age adjusted model (p < 0.05).

Table 5 shows that education is significantly associated with self-rated health after adjustment for age among women in 18 countries [20]. After additional adjustment for emotional support these associations are only slightly attenuated. There is a reduction of more than 10% in Belgium and Israel. Moreover, control for emotional support leads to loss of significance in Ireland and Israel. Taking results in Table 5 together, the relationship between education and self-rated health is hardly mediated by emotional support among women in most countries.

**Table 5:** Education (upper secondary or higher) and good or very good self-rated health: odds ratios (OR)* (95% confidence interval), women, European Social Survey, weighted (n = 19,001)

Country	Adjusted for age	Adjusted for age and emotional support	Change (%)**
Austria	*1.71 (1.25–2.37)*	*1.67 (1.20–2.30)*	-6
Belgium	*2.09 (1.43–3.04)*	*1.97 (1.35–2.90)*	-11
Czech Rep.	*2.55 (1.43–4.53)*	*2.39 (1.33–4.29)*	-10
Denmark	*2.09 (1.37–3.20)*	*2.11 (1.38–3.24)*	+2
Finland	*1.70 (1.19–2.42)*	*1.68 (1.18–2.40)*	-3
France	*2.17 (1.55–3.05)*	*2.15 (1.53–3.01)*	-2
Germany	*1.97 (1.39–2.80)*	*1.93 (1.36–2.76)*	-4
Greece	*2.30 (1.68–3.16)*	*2.31 (1.68–3.16)*	+1
Hungary	*2.84 (2.02–3.99)*	*2.82 (2.00–3.97)*	-1
Ireland	*1.48 (1.03–2.15)*	1.43 (0.98–2.07)	-10
Israel	*1.56 (1.05–2.32)*	1.45 (0.97–2.19)	-20
Italy	1.33 (0.91–1.96)	1.33 (0.90–1.95)	
Luxembourg	*1.54 (1.07–2.21)*	*1.50 (1.04–2.17)*	-7
Netherlands	1.10 (0.85–1.46)	1.09 (0.82–1.45)	
Norway	*1.85 (1.21–2.82)*	*1.85 (1.20–2.80)*	0
Poland	*2.42 (1.77–3.31)*	*2.33 (1.70–3.19)*	-6
Portugal	*2.29 (1.48–3.53)*	*2.26 (1.46–3.49)*	-2
Spain	1.28 (0.88–1.86)	1.25 (0.86–1.81)	
Sweden	*1.69 (1.20–2.38)*	*1.65 (1.17–2.37)*	-6
Slovenia	*2.30 (1.56–3.40)*	*2.23 (1.51–3.31)*	-5
Switzerland	*2.47 (1.65–3.70)*	*2.42 (1.61–3.62)*	-3
UK	1.25 (0.91–1.72)	1.23 (0.90–1.70)	

* Statistically significant OR (p < 0.05) are italicised.

** Percentage change in OR in age adjusted analyses compared with OR in age and emotional support adjusted analyses. Calculated by using:

([OR_age adjusted_-OR_age and emotional support adjusted_]/[OR_age adjusted_-1])*100. Percentage change is displayed when OR is statistically significant in the age adjusted model (p < 0.05).

## Discussion

This study has examined the associations between education, emotional support and self-rated health in 22 European countries. Multiple logistic regression analyses show that emotional support is positively associated with education. However, the magnitudes of these relationships are not consistent. They vary according to the country considered, and even if there are significant effects for one gender, these may not always be reproduced for the other of the same country. Former studies have found that higher levels of social support are related to higher occupational positions [11-13] and higher income levels [14]. However, the association between education and emotional support has not yet been examined in detail, especially not in a comparative perspective.

Moreover, our results confirm the thesis that emotional support is positively associated with self-rated health [3]. However, this association does not seem to be an invariant phenomenon as we found gender and country differences. Gender differences are not consistent, i.e. in some countries association is stronger among women while in other countries health benefits are larger among men. Research on the relationship between social support and health has suggested several pathways linking social support to health [4]. Firstly, social support might increase the likelihood that individuals engage in health promoting behaviours or refrain from health damaging ones. Evidence suggests that, in general, social support is inversely related to health damaging behaviours [4]. In addition, social support may influence help-seeking behaviour. Secondly, social support is likely to work through psychological factors, as social support has been found to be associated with self-efficacy [29], coping-effectiveness [30], and depression [5]. Thirdly, lack of sufficient social support acts as a chronic stressor, resulting in the accelerated ageing of the human organism [31]. There is also growing evidence for neuroendocrine changes and alterations in immune response induced by a lack of social support [4].

Finally, our results show that emotional support explains little of the educational differences in self-rated health among women and men in most European countries. Psychosocial factors like emotional support are often mentioned when possible explanations for health inequalities (including educational health inequalities) are discussed [28]. So far, only a few studies have systematically examined, whether the association between social inequality and health is mediated through social relationships. British studies found that social support is not a major influence in explaining employment grade differences in coronary heart disease, depression, and physical functioning [16,32]. Similarly, the education gradient in coronary heart disease was essentially unchanged after adjustment for social support among Swedish women [17]. A comparative study conducted in Germany and the United States showed that the mediating effect of social relationships (emotional support and social contacts) on the association between socio-economic status and health (self-rated health, depression and functional limitations) among the aged is weak [9].

Several limitations of this study need to be considered. Firstly, this was a cross-sectional study, and it was subject to the problem of common method variance as both the independent and the dependent variables were based on self reports. Thus, no causal inference can be drawn concerning the association of education, emotional support and health. Secondly, response rates differ between the 22 countries with response rates below 50% in the Czech Republic, France, Italy, Luxembourg, and Switzerland [19,20]. If our estimates are sensitive to response rates, the comparability of the estimates for different countries will be reduced. Results from survey research indicate that response rates are lower in lower socio-economic groups and in less healthy people. This could imply that non-response might lead to underestimation of the associations analysed here [25]. It can be assumed that this underestimation is larger in countries with a low response rate. A third methodological problem relates to the use of education as an indicator of socio-economic position. The distribution across the levels of education is skewed, and this skewness varies between cohorts and countries (see Table 1). As we used an educational classification of only two groups (lower secondary or less and upper secondary or higher), estimates of inequalities to some extent are crude. We decided to dichotomise education instead of using more categories for the sake of clearness, and due to the small number of cases in some countries. A fourth methodological problem relates to the use of self-rated health as health indicator, especially in a cross-national study. Respondents from different countries and cultures may not only have different reference levels of health, but response categories may also have different connotations [33]. Although self-rated health has been found to be an appropriate indicator for comparative studies on social variations in health [20,24,25,34,35], it remains to be established whether similar country differences will be found if another health indicator is used. This holds especially for the association between emotional support and health as there is a lack of respective international studies [12]. Finally, we used a simple measure of social support based on only one question assessing the availability of a confidant with whom one can discuss intimate and personal matters. However, as Peggy Thoits stated in 1995, this indicator of perceived emotional support has been found to be "(...) the simplest and most powerful measure of social support (...)" [6, p. 64]. It is one task of future studies to explore whether our results can be replicated when other indicators for social support (e.g. instrumental support) are used. In terms of comparative research on instrumental support it is reasonable to take variations in institutional factors such as differing general welfare systems or differing health care systems into account. Institutional factors are also relevant for interpreting the differing associations of emotional support with health in different countries as the presence or absence of emotional support may have different implications in countries with a developed welfare system as opposed to countries with a less developed welfare system. Therefore, we consider it an important future task to link welfare state research with comparative epidemiological research on health effects of social support.

The methodological limitations are balanced by several strengths. The European Social Survey dataset used here comprises a number of European countries and therefore offers the opportunity to give a comprehensive overview of the association between education, emotional support and health in Europe. The survey was conducted on the basis of a vigorously controlled study protocol, including standard procedures of translating the measures into different languages and of collecting and controlling data [19].

## Conclusion

Association between education and social support indicates the necessity to consider socio-economic factors in research on health effects of social support. Consideration of such factors that are part of the larger macrosocial context in which network and support structures are formed and sustained has been lacking in all but a small number of studies and is almost completely absent in studies of social support influences on health [4]. Consideration of the marcosocial context might also be important for social support interventions. Most of these interventions were restricted to the individual level and have not succeeded in changing health outcomes [12]. Moreover, variations across countries indicate that societies may differ in the value they place on social bonds. In this regard, cross-national studies are essential if we are to understand whether social support generally is an important determinant of population health.

## Competing interests

The author(s) declare that they have no competing interests.

## Authors' contributions

OvdK was responsible for analysing and interpreting the data and writing the article. SG contributed to writing the article and provided feedback on drafts. Both authors have read and approved the final manuscript.

## Pre-publication history

The pre-publication history for this paper can be accessed here:


